# Full‐Body Versus Local Wipe Disinfection With Chlorhexidine Gluconate: No Difference in Surgical Site Infection After Acute Hip Fracture Surgery

**DOI:** 10.1111/iwj.71046

**Published:** 2026-09-20

**Authors:** Eila M. Sterner, Henrike Häbel, Gunnar V. Edman, Wilhelmina Ekström

**Affiliations:** ^1^ Department of Molecular Medicine and Surgery Karolinska Institutet Stockholm Sweden; ^2^ Department of Trauma, Acute Surgery and Orthopedics Karolinska University Hospital Stockholm Sweden; ^3^ Department of Learning, Informatics, Management and Ethics Karolinska Institutet Stockholm Sweden; ^4^ Research and Development Norrtälje Hospital Norrtälje Sweden; ^5^ Department of Clinical Sciences Danderyd Hospital, Karolinska Institutet Stockholm Sweden

## Abstract

Preoperative skin disinfection is essential to reduce surgical site infection. This study compared full body with local skin disinfection and examined additional patient related factors that could influence surgical site infection. This non‐randomized intervention study, conducted over two consecutive periods, included patients with a hip fracture who received preoperative full‐body disinfection with 4% chlorhexidine gluconate or local skin disinfection of the hip using 2% chlorhexidine gluconate, with 90 days follow up. Postoperative dressing change was documented. 844 patients were included in the study; 556 (65.7%) patients were treated with full body disinfection and 288 patients (34.1%) with local wipes disinfection. Fourteen patients (1.7%) suffered from a surgical site infection, eleven patients in the full body disinfection group, and three patients with local wipes disinfection without any significant difference found between the groups. Seven (50%) patients with infection had the dressing changed < 48 h. Chronic kidney disease was significantly associated with local infection (*p* = 0.005). Local preoperative skin disinfection with a washcloth showed no increased risk of wound infection in patients with hip fracture compared to full body disinfection, indicating comparable effectiveness. Impaired kidney function may increase susceptibility. Documentation gaps highlight the need to evaluate adherence to dressing routines to strengthen postoperative wound care. Chlorhexidine gluconate, Full body skin disinfection, Hip fracture, Local wipes disinfection, Surgical site infection.

## Key Points

- Skin disinfection alone cannot account for persistent SSI; both intrinsic and extrinsic risk factors must be systematically addressed.
- Early identification and close monitoring of patients with established chronic kidney disease, as well as those at risk of developing acute kidney injury after surgery, are pivotal for reducing SSI risk and ensuring optimal postoperative management.
- The findings of hypoalbuminemia and anemia may contribute to SSI risk and transfusion requirements, highlighting the importance of nutritional and hematological assessment.
- Strict adherence to clinical routines and current guidelines is essential to safeguard patient safety, strengthen clinical judgment, and minimize dropout rates in research.
- Comprehensive and accurate documentation, including BMI and wound care details, is indispensable for ensuring research validity and maintaining patient safety in practice.

## 1 Introduction

Surgical site infection (SSI) is a common complication and contributes to negative outcomes for patient safety, patients' quality of life and health care organizations [1, 2, 3]. Postoperative infections are one of the three most common complications according to the Public Health Authority of Sweden based on the European Centre for Disease Prevention and Control Point Prevalence survey 2023 [4], along with pneumonia and urinary tract infection. Elderly people undergoing surgery due to a hip fracture are particularly vulnerable group with significant risk of SSI [5]. Underlying comorbidities, such as cardiovascular diseases [6], chronic kidney disease (CKD) [7] and acute kidney injury (AKI) [8, 9], can have an impact on development of SSI and worsen recovery.

Full‐body disinfection (FBD) is a well‐known procedure for prevention of SSI on patients planned for elective surgery [10, 11] and thus, a method used for patients with hip fractures. Moving a patient with an unfixed hip fracture can be painful, and there is a significant risk that the fracture may not remain stabilized during the transfer between the bed and the shower gurney. A previous study has shown that local disinfection with chlorhexidine did not differ from FBD regarding SSI prevention and contributed to less pain due to fewer movements for the patient [12]. While chlorhexidine does achieve high antiseptic concentrations with repeated showers [10] or with impregnated wipes [13], its direct impact and benefit on reducing SSI remain uncertain [14]. A simplified process for preoperative local disinfection for patients with hip fractures may be as effective as FBD.

The incidence of SSI varies from 1% to 10% due to fracture type, surgical methods and follow‐up protocol [2, 15, 16, 17]. Intrinsic and extrinsic factors can have an impact on outcome after hip fracture surgery [18, 19] such as age, malnourishment, AKI and different co‐morbidities [9, 17, 20, 21]. Extrinsic factors may relate to different types of surgical procedure and implant, surgical time, risk of blood loss, operating room environment, timing for antibiotic prophylaxis and preoperative skin preparation [15, 17, 21, 22, 23]. The timing for changing of sterile wound dressings postoperatively remains a concern, as early removal continues to be prevalent in clinical practice [24]. The goal is to have undisturbed wound healing for at least 48 h [25, 26, 27].

In a previous study, chlorhexidine gluconate (CHG) was tested to reduce the endogenous flora. Disinfections on Day 1, 2, and 5 were performed [28]. The result showed that the antiseptic effect increased with every double disinfection up to Day 5; thereafter, there was no significant difference. Therefore, at least three double disinfections were recommended from start based on the residual of antimicrobial effect [28]. More recent studies indicate that two disinfections, each with a pause to rinse, have been demonstrated to reduce contamination on the skin of patients undergoing elective surgery [10, 13]. One wash with CHG does not provide sufficient concentration on the skin to reduce the skin flora since CHG products require multiple applications [21, 29]. Preoperative skin antisepsis involves several established antiseptic agents, and chlorhexidine (CHG) is not the only option. Alcohol‐based povidone–iodine and other iodine‐based combinations are widely used in orthopaedic practice (not in Sweden) and have demonstrated comparable effectiveness to CHG in clean orthopaedic surgery [30, 31]. Orthopaedic reviews further indicate that multiple antiseptic formulations appear clinically equivalent across anatomical sites, underscoring the need for additional orthopaedic‐specific studies [32]. It is also not entirely risk‐free to use CHG since severe allergic reactions such as anaphylactic shock, skin irritation on sensitive skin, and risk for corrosive eye injuries on contact have been found [33, 34]. It is highly toxic to aquatic life both acutely and long‐term according to the European Chemicals Agency's database [35]. The selection of antiseptics, determining the optimal CHG concentration, and addressing side effects as well as antiseptic resistance remain critical issues that must be resolved [36]. Despite the risks, FBD with CHG is recommended in Sweden and described in the Swedish Handbook for Healthcare. However, the need for FBD is assessed individually based on the planned procedure, and exemptions may be made for frail patient groups or when clinical circumstances require adaptation [27].

The aim of this study was to determine the incidence of SSI in patients with hip fracture who underwent either FBD with 4% CHG on a shower gurney or local skin disinfection of the hip using 2% CHG impregnated wipes (LWD) before surgery. The secondary aim was to identify potential risk factors associated with SSI.

## 2 Material and Methods

### 2.1 Study Design, Settings

This non‐randomized intervention study was performed from the years 2014–2018 on patients admitted with a verified acute hip fracture according to the Classification of Disease and ICD—10 codes. The study was conducted at two physically separate university hospitals within Stockholm County Council, Sweden. Patients were admitted either to an orthopaedic or a geriatric ward, according to the hospital catchment area corresponding to their home address.

### 2.2 Patient Characteristics

Patient data was obtained from the hospital administrative care systems, electronic health record (EHR), the surgery planning system, and from the developed study protocol. No changes to the surgical routine at the operation theatre regarding skin preparation, time for antibiotics, wound dressing, and use of warming blankets or liquids were performed.

Patient characteristics such as age, sex, ASA classification, cognitive impairment, diabetes mellitus, hypertension, heart, lung, kidney disease, fracture type and use of nicotine were recorded. Body Mass Index (BMI) score at admission was used to assess risk of malnutrition [37, 38]. Laboratory values including albumin and creatinine before surgery and haemoglobin before and after surgery were recorded, as well as blood transfusion requirements postoperatively. Antibiotic prophylaxis, surgical method, time to surgery, length of stay, mortality within 1 year after hip fracture, SSI within 30 and 90 days after surgery and dressing changes within 48 h postoperatively (and reason for changes) were recorded.

SSI was defined after visible observation of an infected wound and confirmed by positive wound culture [39, 40]. The definition was also based on the recommendation from European Centre for Disease Prevention and Control defining SSI as a superficial wound infection that occurs within 30 days of surgery or deep infection within 90 days if implants [4, 21].

### 2.3 Skin Preparation

The study was divided into two consecutive periods.

In the first study period, preoperative disinfection at the ward was carried out with two ready‐to‐use sponges impregnated with 15 mL of a 4% chlorhexidine gluconate solution (FBD). This solution is water‐based and contains no alcohol and follows the national recommendations [27]. The patients were moved from their beds to a specific shower gurney. This procedure required 3–4 staff members to support the patients. The patient was gently repositioned from side to side to allow thorough washing of the entire body. The CHG solution remained on the skin for a few minutes, reflecting routine clinical practice. The exact contact time was not measured, as the procedure is performed swiftly adapted to minimize patient discomfort, exposure, and the risk of cooling before the skin was thoroughly rinsed with plenty of water. Patients were removed from side to side, so the whole body was washed. The procedure was repeated, and the skin was dried with a clean towel. The hair was washed at one occasion. If the operation was postponed 24 h—the preoperative disinfection was repeated.

**FIGURE 1:**
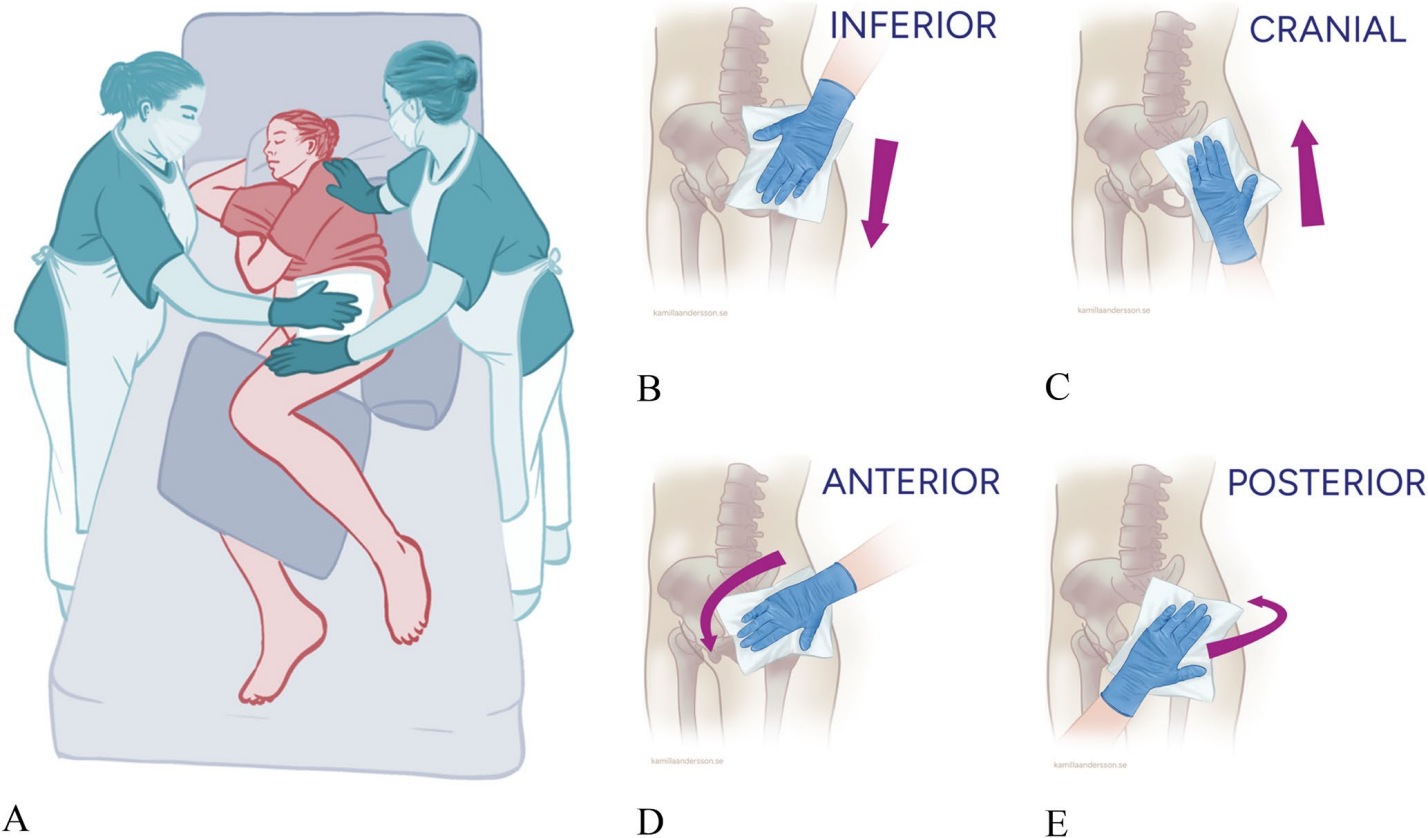
(A–E) Illustration showing the local wipe disinfection procedure and order for disinfection with four wipes.

During the second study period, the patients were treated with four alcohol‐free, ready‐to‐use disposable wipes impregnated with 2% CHG and applied locally according to a specific procedure on the hip with the patient still in bed. Two staff members performed the procedure. The skin disinfection was performed about 1 h before leaving for surgery. The standardized LWD was performed by wiping from the central area at the trochanter major to the peripheral area in one sweep, with no rubbing. Four wipes were used (Figure 1a–e).

If the waiting time for surgery was postponed for 5 h, the procedure was repeated. The patients were provided with clean surgical clothes and bedding after skin preparation in the ward in both periods. No hair removal from the surgical site was performed preoperatively.

### 2.4 Information to Staff

All staff members were informed about the study in four oral briefings, two offered during day shifts and two during night shifts. Staff members were free to attend any of the sessions. The staff were given written information about the study, and two posters, one for each study period, were positioned in each ward as an extra reminder. A control survey was conducted in the second study period to ensure that the staff knew how and in what order the four impregnated wipes should be used. Two research assistants with overall responsibility were appointed in each ward. The supervisor was in close contact with the research assistants during the whole study period. The research assistants received information about the study and how to proceed by contacting the supervisor if questions arose. The staff were responsible for information to patients about preoperative disinfection before surgery.

### 2.5 Clinical Routines for Antibiotics and Dressings

The first antibiotic dose was given to each patient within the 30‐min interval before the surgery. Patients who were planned for osteosynthesis were treated with Cefuroxime (1,5 g × 3) injections and patients planned for arthroplasty received Cloxacillin (2 g × 3) injections and if penicillin allergy Cefotaxime (2 g × 2) or Clindamycin (600 mg × 2) was prescribed according to guideline [41, 42].

The wound dressings were applied postoperatively in a theatre under sterile conditions. According to clinical and national guidelines, wound dressings should be closed for at least the first 48 h postoperatively [25, 26, 27]. The sterile dressings were permitted and removed earlier if signs of wound rupture, infection, or bleeding were evident, and always changed with aseptic technique [27]. The reason for the dressing change was documented in the EHR.

### 2.6 Study Population

In total there were 1218 patients available for the two study periods. Patients with a pathological hip fracture, a fracture in conjunction with previous osteosynthesis or prosthesis, and a hip fracture after multiple trauma were excluded as well as patients admitted with a new hip fracture on the other side during the study period. To ensure a homogenous study population, the three most common types of hip fractures (S72.0—S72) were selected. Fractures on the femoral shaft (S72.3) and the distal femur (S72.4) were excluded as these are frequently caused by high‐energy trauma and require more time‐consuming surgical interventions. Since prolonged operative duration (particularly exceeding 100–120 min) is a well‐documented independent risk factor for postoperative surgical site infections [43], the selection was limited to minimize the risk of bias regarding complication rates.

Patients who received care in departments other than orthopaedics or geriatrics prior to surgery were excluded. Additionally, patients who were temporarily transferred to another healthcare facility due to unrelated medical conditions before surgery were also excluded. Patients who died before 30 days follow‐up or were cared for outside the actual health care region or abroad after surgery were excluded due to follow‐up not being possible. After exclusion, a total of 844 patients remained who fulfilled the inclusion criteria.

### 2.7 Ethics Approval and Consent to Participate

The study was conducted in accordance with international research standards and Swedish National Regulations and was approved by the Regional Ethics Committee of Stockholm (Dnr: 2013/1897–32).

### 2.8 Statistical Method

JASP Team (2024). JASP (Version 0.19.3) (Computer software) and SPSS (IBM Corp. (2017). IBM SPSS Statistics for Windows (Version 28.0)) were used.

Continuous variables were presented as mean and standard deviation (SD) or median and interquartile range (IQR = 3rd quartile—1st quartile) when more appropriate due to a skewed distribution. Variable distributions were visually inspected by using histograms to determine the presence of skewness. Categorical variables were summarized as frequencies (proportions). Patient characteristics were compared between the two disinfection groups, FBD and LWD. SSI incidence was studied by comparing characteristics between patients with and without SSI. Differences in continuous variables between groups were tested using Student' *t*‐test or Mann–Whitney U test when more appropriate due to a skewed distribution. Differences in proportions were tested using the Pearson χ^2^‐test or Fischer's exact test when an expected cell frequency was below 5 and an exact test was, hence, more appropriate.

To identify potential risk factors for SSI, a multivariable logistic regression model was fitted including the known confounders age and sex as well as all variables that showed a weakly or strongly statistically significant (*p* < 0.1) difference in SSI incidence groups in the univariate analyses. Because of severely skewed distributions for some variables, all independent variables were dichotomized according to unbiased criteria such as median (see Supplementary S1 for more details). Adjusted odds ratios (95% confidence intervals (CI)) were reported to illustrate the strength of association. Missing values were assumed to be missing at random, and no imputations were made. Two‐sided *p*‐values were reported and a *p*‐value < 0.05 was considered significant. Due to the explorative nature of this study, no corrections for multiple testing were made.

## 3 Results

Of the 844 patients included in the analysis, there were 556 patients (65.7%) treated with FBD with 4% CHG (two sponges) and 288 patients (34.1%) treated with LWD with 2% CHG (four wipes).

**FIGURE 2:**
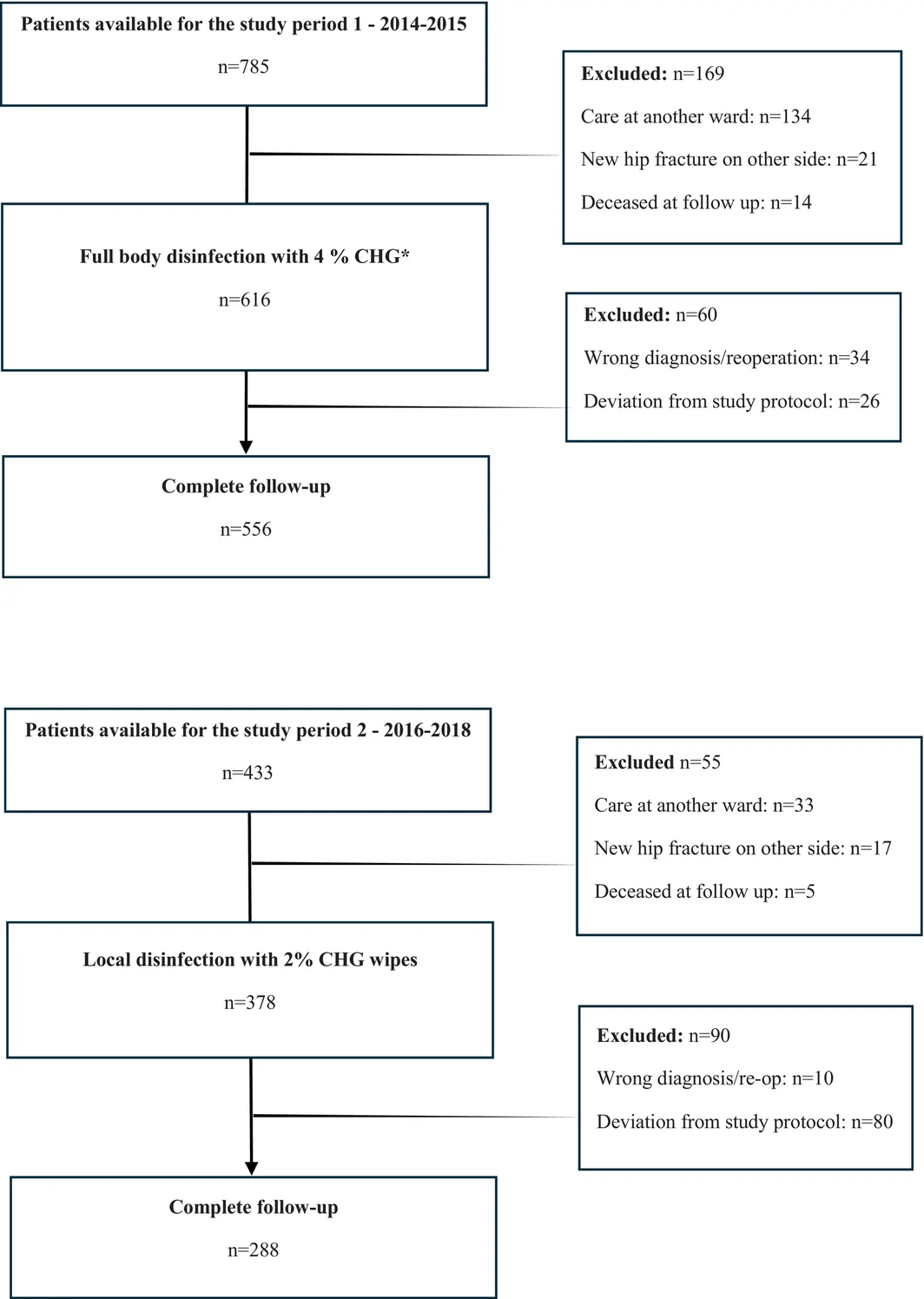
Flowchart showing patients with hip fracture available for study period 1 having preoperative treatment with double full body disinfection or with local wipes in study period 2. Reasons for exclusion are demonstrated. CHG, Chlorhexidin Gluconate.

There were a total of 106 dropouts and different reasons were noted (Figure 2). Among them there were 26 dropouts in the FBD group: two patients with SSI. The disinfection methods used in these two cases were a single CHG disinfection and no disinfection before being admitted to surgery. In the LWD group, there were 80 dropouts and seven patients with SSI. These seven patients were disinfected with a double shower according to standard procedure with 4% CHG as in the FBD group.

There were few significant differences in the demographics between patients in the FBD and LWD groups (Table 1). BMI was assessed at admission in 805 patients (95.4%); score ranged within interval from 15.6–49.8. The BMI score was missing in 21 patients in the FBD group and 18 patients in the LWD group. Approximately three quarters of the patients had an ASA score ≥ 3; 642 (76.1%) patients were distributed to 412 patients in the FBD group and 230 patients in the LWD group.

**TABLE 1:** Characteristics of patients receiving disinfection with either full‐body or local wipes.

Characteristics	Group	Statistics	Disinfection groups		
			Full body	Local wipes	*p*
Age		Mean (SD)	83.6 (8.41)	83.0 (8.52)	0.304^a^
Sex	Female	*n* (%)	381 (68.5)	202 (70.1)	0.631^b^
	Male	*n* (%)	175 (31.5)	86 (29.9)	
BMI		Mean (SD)	23.5 (4.29)	23.4 (4.23)	0.787^a^
ASA class	1–2	*n* (%)	144 (25.9)	58 (20.1)	0.063^b^
	3–4	*n* (%)	412 (74.1)	230 (79.9)	
Type of hip fracture	Femoral neck fracture	*n* (%)	297 (53.4)	152 (52.8)	0.860^b^
	Pertrochanteric fracture	*n* (%)	226 (40.6)	105 (36.5)	0.237^b^
	Subtrochanteric fracture	*n* (%)	33 (5.9)	31 (10.8)	0.012^c^
Comorbidities	Hypertension	*n* (%)	326 (58.6)	154 (53.5)	0.151^b^
	Heart disease	*n* (%)	224 (40.3)	105 (36.5)	0.279^b^
	Cognitive impairment	*n* (%)	148 (26.6)	82 (28.5)	0.566^b^
	Diabetes mellitus	*n* (%)	94 (16.9)	50 (17.4)	0.868^b^
	Chronic obstructive pulmonary disease	*n* (%)	59 (10.6)	48 (16.7)	0.012^b^
	Chronic kidney disease	*n* (%)	42 (7.6)	23 (8.0)	0.823^b^
Laboratory status at admission, before surgery	Haemoglobin (g/L)	Mean (SD)	100.3 (14.57)	98.8 (14.28)	0.158^a^
	Albumin (g/L)	Median (IQR)	32.9 (5.08)	31.6 (5.61)	0.002^d^
	Creatinine (μmol/L)	Median (IQR)	89.0 (73.7)	87.9 (42.3)	0.810^d^
Nicotine	Snuffs or smokes	% (n)	56 (10.2)	34 (11.9)	0.445^b^

*Note:* Statistical methods: ^a^ *t*‐test, ^b^chi‐squared test, ^c^Fisher's exact test, ^d^Mann–Whitney U test.

Abbreviations: ASA, physical status according to American society of anesthesiologists; BMI, body mass index; g/L, gram per litre; IQR, median (interquartile range); SD, standard deviation; μmol/L, micromole per litre.

Femoral neck fracture was the most common fracture type; 449 (53.1%) patients. A total of 1239 comorbidities were registered and several patients had multiple comorbidities. The most common diseases were hypertension; 480 patients, 56.9% in the FBD group and 52.8% patients in the LWD group followed by heart disease in 329 patients, 40.0% in the FBD group and 36.4% in the LWD group. Patients in the LWD group were significantly more affected by chronic obstructive pulmonary disease (*p* = 0.012). An albumin level of < 35 g/L was found in 59% of the patients, median (IQR) 34 (30–36) in the FBD group and 32 (28–38) in the LWD group (*p* = 0.002). High creatinine levels (> 100 μmol/L) were found in 20.7% of patients without having any documented chronic kidney disease and distributed for the FBD group of 109 (67.7%) patients and the LWD group 52 (32.3%) patients. No difference was found in haemoglobin value between the groups. See Table 1 for more details on characteristics.

There were 14 patients (1.7%, 95%, CI 0.8%–2.5%) that suffered from an SSI of which 11 patients were treated with FBD and three with LWD (Table 3). Five patients (FBD *n* = 3; LWD *n* = 2) underwent excisional arthroplasty (Girdlestone, NFG09). Of these five patients; one patient was treated with FBD developed SSI. This procedure was excluded from the statistical model due to the very small number of patients. Ten patients had their infection within 30 days, distributed to eight patients in FBD group and two in LWD group. Four patients had an SSI within 90 days, three in FBD group and one in LWD group. These four patients had a waiting time for surgery > 24 h. No significant differences were found in SSI incidence between the two disinfection groups.

Among all patients with an ASA score ≥ 3, 13 (2.0%) patients had SSI, 10 patients in the FBD and three in the LWD group. Of these 13 patients, 10 patients had a BMI score > 20, eight in the FBD group and two in the LWD group. Three patients with SSI had a BMI score ≤ 20, distributed to two patients in the FBD and one in the LWD group. The single remaining patient with SSI had an ASA score of 2 and a BMI > 20. See Table 2 for more details according to characteristics between disinfection groups and SSI.

**TABLE 2:** Characteristics for patients with hip fracture and occurrence of surgical site infection within 90 days.

Variable	Value label		Surgical sites infection		*p*
			No < 30 days	Yes < 30 days	
Disinfection methods	FBD	*n* (%)	548 (98.6)	8 (1.4)	0.508^b^
	LWD		286 (99.3)	2 (0.7)	
			No < 90 days	Yes < 90 days	
Disinfection methods	FBD	*n* (%)	545 (98.0)	11 (2.0)	0.402^b^
	LWD		285 (99.0)	3 (1.0)	
			No	Yes	
Age ≥ 80 year		*n* (%)	555 (98.1)	11 (1.9)	0.567^b^
Sex	Woman	*n* (%)	572 (98.1)	11 (1.9)	0.568^b^
	Man		258 (98.9)	3 (1.1)	
BMI ≥ 20		*n* (%)	622 (98.1)	12 (1.9)	0.745^b^
ASA	1–2	*n* (%)	201 (99.5)	1 (0.5)	0.207^b^
	3–4		629 (98.0)	13 (2.0)	
Hip fracture					
S72.0 = Femoral neck fracture		*n* (%)	439 (97.8)	2.2 (10)	0.168^a^
S72.1 = Pertrochanteric fracture		*n* (%)	328 (99.1)	3 (0.9)	0.169^a^
S72.2 = Subtrochanteric fracture		*n* (%)	63 (98.4)	8 (1.6)	1.000^b^
Comorbidities					
Cognitive impairment		*n* (%)	226 (98.3)	1.7 (4)	1.000^b^
Diabetes mellitus		*n* (%)	142 (98.6)	1.4 (2)	1.000^b^
Heart disease		*n* (%)	507 (98.4)	1.8 (6)	0.764^a^
Hypertension		*n* (%)	472 (98.3)	1.7 (8)	0.984^a^
Chronic obstructive lung disease		*n* (%)	105 (98.1)	1.9 (2)	0.695^b^
Chronic kidney disease		*n* (%)	61 (93.8)	6.2 (4)	0.018^b^

			Surgical sites infection		
Variable	Value label		No	Yes	
Surgery					
Time to surgery	< 24 h	n (%)	253 (97.7)	6 (2.4)	0.381^b^
	> 24 h		577 (98.6)	8 (1.4)	
Surgical methods					
NFB19 = Hemiarthroplasty		*n* (%)	208 (96.7)	7 (3.3)	0.056^b^
NFB49 = Total hip arthroplasty		*n* (%)	80 (97.6)	2 (2.4)	0.638^b^
NFJ49 = Open reduction internal fixation		*n* (%)	147 (100.0)	0 (0.0)	0.146^b^
NFJ59 = Intramedullary nail		*n* (%)	275 (98.6)	4 (1.4)	0.484^b^
NFJ69 = Sliding hips screw		*n* (%)	116 (100.0)	0 (0.0)	0.237^b^
After surgery					
Haemoglobin after surgery (g/L)		Mean (SD)	100 (14.4)	97 (17.7)	0.464^c^
Transfusion after surgery		*n* (%)	345 (98.0)	7 (2.0)	0.526^a^
Change of bandage < 48 h after surgery		*n* (%)	395 (98.3)	7 (1.7)	0.858^a^
Length of stay at ward in days		Median (IQR)	9.2 (6.0)	10.6 (9.6)	0.932^d^
One year survival after hip fracture surgery		*n* (%)	167 (95.5)	6 (3.5)	0.047^b^

*Note:* Statistical methods: ^a^Chi‐squared test, ^b^Fischer's exact test, ^c^ *t*‐test, ^d^Mann–Whitney U test. Frequencies (proportions) when characteristics were not present are not shown but used in the statistical hypothesis testing. Excision arthroplasty of the hip; Girdlestone procedure (NFG09) are missing—of five patients, one patient has surgical site infection, not in the statistical model because it is too few patients.

Abbreviations: ASA, physical status according to American society of anesthesiologists; BMI, body mass index; FBD, full body disinfection; g/L, gram per litre; LWD, Lokal wipes disinfection; QR, median (interquartile range); SD, standard deviation.

When comparing patient characteristics and presence of SSI were compared, we found no significant difference except regarding chronic kidney disease (*p* = 0.018). In total, 65 patients had chronic kidney disease, and 4 patients (6.2%) had an SSI. Documentation for prescribed or given antibiotics was missing in total for 37 (4.4%) patients; 29 in FBD and eight in the LWD group, respectively. No SSI was found in these groups of patients.

The most common surgical method was treatment with intramedullary nail; in 279 (33.1%) patients, followed by primary hemiarthroplasty in 215 (25.5%). Among these 215 patients with hemiarthroplasty, 7 (3.3%) had SSI compared to 7 (1.1%) among the 629 patients not treated with hemiarthroplasty (*p* = 0.056) (Tables 2 and 3).

**TABLE 3:** Surgical site infections within 90 days by preoperative skin disinfection, fracture type and surgical method.

Hip fracture	Disinfection groups				
		Full body		Local wipes	Total
		With SSI		With SSI	With SSI
	*n*	n (%)	*n*	*n* (%)	
Femoral neck fracture	297	8 (2.7)	152	2 (1.3)	10 (2.2)
Pertrochanteric fracture	226	0.9 (2)	105	1 (0.9)	3 (0.9)
Subtrochanteric fracture	33	3.0 (1)	31	0	1 (1.6)
Total SSI within 90 days					14 (1.7)
	Disinfection groups				
		Full body		Local wipes	Total
		With SSI		With SSI	With SSI
Surgical methods^a^	*n*	n (%)	*n*	*n* (%)	
Hemiarthroplasty	146	5 (3.4)	69	2 (2.9)	7 (3.3)
Total hip arthroplasty	51	2 (3.9)	31	0	2 (2.4)
Open reduction internal fixation	97	0	50	0	0
Intramedullary nail	182	3 (1.6)	97	1 (1.0)	4 (1.4)
Sliding hip screw	77	0	39	0	0
Total SSI within 90 days					13 (1.5)^a^

Abbreviation: SSI, surgical site infection.

^a^ Five patients underwent excision arthroplasty of the hip (NFG09–Girdlestone), including one patient with SSI. This surgical method was not part of the study's analytical framework, and the entire group was therefore excluded from the statistical model.

Patients with SSI had a length of surgery from 61 to 221 min, nine patients in the FBD and three patients in the LWD group. For two patients, the length of surgery was missing. For 297 patients with hemi or total hip replacement who received an arthroplasty, three doses of antibiotics were documented for 236 patients (79.1%), 156 (66.1%) patients in FBD (four with SSI), and 80 (33.9%) patients in LWD group (one with SSI).

In total 352 (41.7%) patients had blood transfusion after surgery. In the FBD group 163 (29.3%) patients had transfusions, and seven (3.3%) among them developed an SSI. No transfusion and SSI were observed in the LWD group. The length of stay varied from 1 day to 34 days and for patients with SSI, 1–20 days in FBD group and 7–9 days in LBD group. SSI may be associated with higher one‐year mortality (*p* = 0.047) (Table 2). Among 173 patients who were deceased after 1 year, six (3.5%) patients had SSI; five patients in the FBD group and one patient in the LWD group. There were 671 (80%) patients who survived 1 year after their hip fracture surgery out of which eight (1.2%) patients had an SSI, six in FBD group and two in LWD group.

There were 402 patients (43.3%) that had a change of wound dressing within < 48 h distributed to 273 (67.9%) in FBD group and 129 (47.3%) in LWD group. Several changes of dressings during hospital stay, not only at discharge, were found in 321 (80.0%) patients, in FBD group 205 (63.9%) patients and in LWD group 116 (36.1%). Reasons given for dressing changes were; dressing full of blood/exudate or loosened in 220 (44.0%), patient caused reasons in 99 (19.8%) cases, or the wound needed inspection, in 83 (16.6%) patients. Among six patients diagnosed with SSI, no documentation was found regarding the appearance of the wound or the wound dressing. For one of these patients, the only recorded intervention was a dressing change upon discharge, despite SSI and with a length of stay for 14 days. Documentation of dressing change was missing in total of 244 (29.0%) distributed for 150 (61.5%) in FBD group and 94 (38.5%) in LWD group. Seven of the patients with SSI had a change of wound dressing within < 48 h, five patients in FBD and two patients in LWD group (Table 2).

In the adjusted analysis (Table 4), chronic kidney disease was statistically significantly associated with SSI (OR 5.632, 95% CI from 1.664 to 19.059, *p* = 0.005) and may be a potential risk factor. Furthermore, some evidence was found that surgery with hemiarthroplasty could be associated with higher odds for SSI (OR 2.88, 95% CI from 0.97 to 8.48, *p* = 0.056) (Table 4). Receiving skin preparation in the wards, sex, and being 80 years or older did not show any association with SSI. Although previous studies have reported such associations, our findings indicate that no relationship could be demonstrated in this cohort.

**TABLE 4:** Logistic regression analysis of the relationship between surgical site infection 90 days after hip surgery and five potential risk factors.

	B	SE	Wald	df	*p*	OR	95% CI for OR	
							Lower	Upper
Received preparation at ward	−0.341	0.333	1.05	1	0.305	0.711	0.370	1.364
Chronic kidney disease at injury	1.728	0.622	7.72	1	0.005	5.632	1.664	19.059
Surgery with hemiarthroplasty	1.056	0.552	3.66	1	0.056	2.876	0.974	8.487
Female	0.736	0.675	1.19	1	0.275	2.088	0.556	7.835
Age ≥ 80	0.394	0.672	0.34	1	0.558	1.483	0.397	5.534
Constant	−5.793	1.417	16.72	1	< 0.001	0.003		

## 4 Discussion

In this study we compared the incidence of SSI in patients with acute hip fractures that had preoperative disinfection with two ready‐to‐use water‐based 4% CHG sponges (FBD) or disinfection with 4 wipes (LWD) on one occasion with 2% CHG on the fractured hip only. The result of our study showed no significant difference for SSI between the two groups. Seven patients with infection had a changed wound dressing < 48 h. CKD was significantly associated with SSI, in line with several other studies [44, 45]. In addition, there was a predominance of elderly and female patients [46].

Postoperative wound infections are still a problem but are largely preventable with proper adherence and compliance with developed guidelines [47, 48, 49, 50]. Presence of SSI within 90 days shows no significant difference between the groups and the frequency of SSI in our study is similar reported in other studies [2, 17]. The study focused on the most common acute hip fractures (ICD—10, S72.0—S72.3), which share similar pathophysiology and standardized surgical procedures. Other femoral and hip fracture types differ substantially in trauma mechanism, comorbidity burden and surgical management and were therefore not included [15, 16, 51, 52].

Antibiotic prophylaxis is critical to avoid SSI in orthopaedic surgery. Optimal timing (e.g., 30 min before incision) and correct dosage are essential to efficacy and minimizing resistance [42, 53]. According to the Swedish national care program for hip fracture, three doses are given for prosthesis in hip fracture, but the doses should be reduced in cases of severe kidney failure. In osteosynthesis, 1–2 doses are given, with the first dose administered before the start of the operation [41]. Our finding that documentation gaps were present in 37 patients' medical records (4.9%) likely reflects system‐level inconsistencies rather than true deviations from recommended antibiotic prophylaxis routines, as treatment may have been administered but documented elsewhere or not transferred into the EHR. Procedures exceeding 60 min are associated with increased SSI risk [15, 54, 55, 56] and in our study all patients with SSI had a surgical length of 62–221 min. Time to surgery may not be associated to SSI [57] but focus on reducing waiting time to surgery for high‐risk patients with comorbidities [58] is important. Delayed surgery for > 4 days may reduce the nutrition intake and impair compliance with preoperative skin disinfection, and this has been reported as an independent risk factor for SSI [43, 53, 59, 60]. Guidelines by the Association of Anaesthesia recommend access to surgery within less than 36 h [61]. In Sweden the recommendation is access to surgery as soon as possible and that most medical conditions that prevent urgent surgery should be treatable and improve within 24–36 h [41]. In our study nine patients with SSI had surgery between 26 and 48 h and had a length of stay for 4–20 hospital days. However, a randomized study showed that there is no reason to pursue extremely short time to surgery, considering mortality or complication rates [62].

Intrinsic factors like low preoperative albumin and haemoglobin levels are linked to delayed wound healing, malnourishment, impaired recovery capacity and higher complication rates [40, 63, 64]. The combination of low haemoglobin and albumin levels, together with a high ASA score, may indicate frailty, and increased risk of adverse outcomes [5, 65, 66, 67, 68, 69]. In our study 76.1% of patients had ASA score of 3–4. We found that 16.9% of patients lacked albumin data, and 50% had levels below threshold (< 3 5 g/L). We also found that five patients with SSI also had an albumin level ≤ 35 g/L. Although hypoalbuminemia was associated with SSI, albumin was not routinely monitored or corrected during our study, reflecting standard practice in which albumin is not consistently ordered, repeated, or acted upon in short‐stay orthopaedic care. Consequently, our dataset does not allow conclusions about the effect of albumin correction on SSI risk. In our study 41.7% of patients had a blood transfusion and 7 of these patients had a SSI. Low haemoglobin and need for blood transfusion postoperatively correlate with advanced age and prolonged surgery [48, 70, 71]. Low levels of albumin and haemoglobin were observed, alongside an increased need for transfusion in patients with SSI.

Undetected malnutrition can increase infection risk [72, 73] and lead to the risk of missed care [74]. BMI was documented in over 95% of patients, but the accuracy is questionable. Accurate calculation of BMI is essential for identifying well‐nourished patients and ensuring safe clinical decision‐making [75]. Obtaining a reliable weight measurement is particularly challenging in patients with hip fractures due to pain and the risk of further injury associated with movement, especially in the absence of appropriate weighing equipment. Factors such as patient discomfort and the time pressure faced by nursing staff may lead to both under‐ and overweight estimation rather than direct measurement [76]. Alarmingly, studies have shown that only 13.5%–55% of hospitalized patients have their body weight properly recorded [77]. Notably, female patients are more likely to have their weight inaccurately estimated [77] further underscoring the need for systematic and reliable weighing procedures.

Current routines for preoperative skin preparation are largely developed for elective surgery rather than acute conditions except for a recent study [12] that shows that local disinfection of the hip was as effective as full‐body disinfection in preventing SSIs, potentially sparing patients' significant pain. CHG should be prescribed by physicians when preoperative use is indicated considering the chosen surgical method and the associated risk of SSI [27]. Copanitsanou et al. [78] reported that SSIs are largely preventable, and that wound healing may be more closely associated with aseptic practices and surgical technique than with other risk factors alone.

Preoperative skin preparation is a critical component of infection prevention, particularly in elderly patients whose skin is structurally fragile. For microbial defence and supporting wound healing, skin products with a pH around 4.5 are needed with a sponge [79, 80]. Routine scrubbing with antiseptics like 4% CHG antiseptic ready‐to‐use sponges (pH 5.5–7.0‐ Fresenius Kabi) may disrupt the skin's acidic barrier [79, 80]. Evidence suggests that non‐rinse CHG 2% wipes may offer a gentler alternative, preserve bactericidal efficacy left on the skin to be air dried [81, 82] and minimise skin damage from scrubbing with a sponge. Even though CHG is a commonly used antiseptic product in healthcare and suitable bactericidal [10, 13] it needs to be used with care as it is highly toxic and can lead to allergic reactions [33, 34] and can be harmful for aquatic organisms [35].

We found that CKD was an independent predictor of SSI (*p* = 0.005). This association is biologically plausible, as CKD emerged as an independent predictor of SSI, consistent with evidence that renal impairment reduces immune function, alters drug handling, increases postoperative infection risk and impairs wound healing [83, 84]. In our cohort, nearly 20% of patients had elevated creatinine despite no documented CKD, suggesting that under‐recognised renal dysfunction may contribute to SSI risk. Several perioperative factors may contribute to transient or unrecorded renal dysfunction in this population, including advanced age and surgical delay [8, 85], prolonged fasting and insufficient fluid intake [60, 86] and the increased metabolic stress associated with hip‐fracture care. Delayed surgery often results in extended periods without adequate nutrition or hydration, which may exacerbate frailty‐related deficits and increase SSI risk [87]. Ensuring adequate preoperative fluids and nutrition is therefore important to avoid renal complications [72, 88].

Surgical wound care is predominantly nurse‐led, often shaped by local routines rather than strong clinical evidence and interprofessional collaboration [50]. Best practice recommends keeping sterile dressings applied in the operating room intact for at least 48 h of post‐surgery, unless signs of infection, bleeding, or wound rupture are present [27, 89]. Most surgical wounds establish a sufficient barrier within this timeframe [21, 89, 90], though larger wounds may require more time. Minimizing dressing changes supports undisturbed wound healing and reduces the risk of contamination [89, 91, 92]. In our study, dressings were changed within 48 h for 43.3% of patients and reasons varied. We could not find any documentation in 28.9% in the EHR regarding surgical wound appearance. The observed variation in dressing‐change timing likely reflects documentation gaps rather than deviations from the study protocol, as wound care was occasionally recorded outside the EHR. Incomplete records on wound appearance, dressing rationale, adherence to guidelines can compromise patient safety and continuity of care [93, 94, 95, 96]. The limited research on acute wound assessment and documentation underscores the need for further studies to inform surgical nursing practice is essential [97, 98]. Poor record keeping has the potential to lead to poor outcomes of nursing care activities and patient safety [99].

### 4.1 Limitations and Strengths

There were deviations from the study design and treatment protocol that may have affected the results. Workload, stress, and ethical concerns when moving a patient with pain may have affected the outcomes. Personnel turnover may also have affected compliance with the study protocol. The study was performed for several years but the same disinfection methods are still in use. Two different periods at two different wards in two hospitals can affect compliance with the study design. The study is based on the EHR and data from a specific study protocol with questions on what information is needed to be documented. Missing or wrong documentation may affect data and the reliability of adjustment for confounders.

Despite the lack of a statistically significant difference between the two disinfection methods, this study provides important insight: in vulnerable patient groups, factors beyond the choice of disinfection method may influence the risk of SSI. Moreover, studies conducted by frontline healthcare staff in real‐world clinical settings remain scarce yet crucial for understanding and improving everyday patient care.

## 5 Conclusion

This study compared the incidence of surgical infections in patients with hip fracture having preoperative disinfection with two double showers or local disinfection on the hip only with wipes, showing no significant difference for SSI between the two groups. The frequency of postoperative wound infection (1.7%) aligns well with previous reports This could suggest that skin disinfection alone isn't the only explanation for wound infections. Using wipes may reduce patient's discomfort and staff workload. Adherence to wound dressing guidelines appears important as seven patients with infection had a changed wound dressing < 48 h without documented indications. Chronic kidney disease was significantly associated with the rate of local infection indicating that renal function, even AKI, ought to be monitored by staff. Further RCTs are warranted to identify optimal skin disinfection strategies with focus on SSI.

## 6 Summary

- Skin disinfection alone cannot account for persistent SSI; both intrinsic and extrinsic risk factors must be systematically addressed.
- Early identification and close monitoring of patients with established chronic kidney disease, as well as those at risk of developing acute kidney injury after surgery, are pivotal for reducing SSI risk and ensuring optimal postoperative management.
- The findings of hypoalbuminemia and anaemia may contribute to SSI risk and transfusion requirements, highlighting the importance of nutritional and haematological assessment.
- Strict adherence to clinical routines and current guidelines is essential to safeguard patient safety, strengthen clinical judgement, and minimize dropout rates in research.
- Comprehensive and accurate documentation, including BMI and wound care details, is indispensable for ensuring research validity and maintaining patient safety in practice

## Funding

The authors have nothing to report.

## Ethics Statement

The study was conducted in accordance with international research standards and to Swedish National Regulations and was approved by the Regional Ethics Committee of Stockholm [Dnr: 2013/1897‐32].

## Conflicts of Interest

The authors declare no conflicts of interest.

## Supporting information


**Supplementary S1** All independent variables were dichotomized according to different criteria.

## Data Availability

Research data are not shared.
