# Supplementary material for: Full‐Body Versus Local Wipe Disinfection With Chlorhexidine Gluconate: No Difference in Surgical Site Infection After Acute Hip Fracture Surgery
Source: Int Wound J. 2026 Sep 20;23(9):e71046. doi: 10.1111/iwj.71046 (PMC13590210; doi:10.1111/iwj.71046)
Supplement: Supplementary file 1 [file IWJ-23-e71046-s001.docx]

**SUPPLEMENT /APPENDIX**

All independent variables were dichotomized according to different criteria.

| **Independent variables** | **Dichotomized**  Y=yes; N=no | **Criteria** | **Missing**  **(FBD/LWD)** |
| --- | --- | --- | --- |
| Full body disinfection (FBD) | Y/N | As routine | 106 (26/80) |
| Local wipes disinfection (LWD) | Y/N | Intervention |  |
| Age | Y/N | >80 |  |
| Sex | Y/N | Man/female |  |
| BMI | Y/N | ≥20 | 15 (10/5) |
| ASA | Y/N | 1-2, 3-4 |  |
| Diagnose, ICD-10 codes | Y/N | 1-Femoral neck fracture (S720/7200)  2-Per trochanteric fracture (S72.10/11)  3-Subtrochanteric fracture (S72.20/21) |  |
| Hemoglobin (g/L) | Y/N | At admission, mean; SD |  |
| Albumin (g/L); | Y/N | At admission, <35, >35 (median, IQR) | 143 (88/55) |
| Creatinine (µmol/L); | Y/N | At admission, >100 (median, IQR) |  |
| Cognitive impairment | Y/N | At admission |  |
| Diabetes | Y/N | At admission |  |
| Heart disease | Y/N | At admission |  |
| Hypertension | Y/N | At admission |  |
| Chronic obstructive pulmonary disease | Y/N | At admission |  |
| Chronic kidney disease | Y/N | At admission |  |
| Surgery | Y/N | <24, >24 |  |
| Antibiotic | Y//N | All surgical methods | 37 (29/8) |
| Antibiotic, three doses for | Y/N | 1-Hemiarthroplasty (NFB19)  2-Total Hip Arthroplasty (NFB49) | 2 (2/0)  2 (2/0) |
| **Supplement continued next page** | | | |

| **Supplement (continued)** |  |  |  |
| --- | --- | --- | --- |
| **Independent variables** | **Dichotomized**  Y=yes; N=no | **Criteria** | **Missing**  **(FBD/LWD)** |
| Surgical methods | Y/N | 1-Hemiarthroplasty (NFB19)  2-Total Hip Arthroplasty (NFB49)  3-Open reduction internal fixation (NFJ49)  4-Intramedullary nail (NFJ59)  5-Sliding hips screw (NFJ69)  6-Excision arthroplasty of the hip (NFG09)^a^ | 5 (3/2) |
| Hemoglobin (g/L) | Y/N | After surgery, mean; SD |  |
| Transfusion | Y/N | After surgery |  |
| Mortality | Y/N | 1 year after hip fracture |  |
| Length of stay |  | Median (IQR) to discharge |  |
| Dressing change | Y/N | <48 hours | 244 (150/94) |
|  |  |  |  |

^a^ NFG09 -not included, statistically
